# Comparative analysis of mitochondrial genomes reveals marine adaptation in seagrasses

**DOI:** 10.1186/s12864-022-09046-x

**Published:** 2022-12-03

**Authors:** Jun Chen, Yu Zang, Shuo Liang, Song Xue, Shuai Shang, Meiling Zhu, Ying Wang, Xuexi Tang

**Affiliations:** 1 College of Marine Life Sciences, Ocean University of China, Qingdao, Shandong China; 2 Key Laboratory of Marine Eco-Environmental Science and Technology, First Institute of Oceanography, Ministry of Natural Resources, Qingdao, Shandong China; 3 Laboratory for Marine Ecology and Environmental Science, Qingdao National Laboratory for Marine Science and Technology, Qingdao, Shandong China

**Keywords:** Seagrass, Mitochondrial genome, Comparative genomics, Adaptive evolution

## Abstract

**Background:**

Seagrasses are higher marine flowering plants that evolved from terrestrial plants, but returned to the sea during the early evolution of monocotyledons through several separate lineages. Thus, they become a good model for studying the adaptation of plants to the marine environment. Sequencing of the mitochondrial (mt) genome of seagrasses is essential for understanding their evolutionary characteristics.

**Results:**

In this study, we sequenced the mt genome of two endangered seagrasses (*Zostera japonica* and *Phyllospadix iwatensis*). These data and data on previously sequenced mt genomes from monocotyledons provide new evolutionary evidence of genome size reduction, gene loss, and adaptive evolution in seagrasses. The mt genomes of *Z. japonica* and *P. iwatensis* are circular. The sizes of the three seagrasses (including *Zostera marine*) that have been sequenced to date are smaller than that of other monocotyledons. Additionally, we found a large number of repeat sequences in seagrasses. The most abundant long repeat sequences were 31–40 bp repeats. Our study also found that seagrass species lost extensive ribosomal protein genes during evolution. The *rps7* gene and the *rpl16* gene of *P. iwatensis* are exceptions to this trend. The phylogenetic analysis based on the mt genome strongly supports the previous results. Furthermore, we identified five positive selection genes (*atp8*, *nad3*, *nad6*, *ccmFn*, and *matR*) in seagrasses that may be associated with their adaptation to the marine environment.

**Conclusions:**

In this study, we sequenced and annotated the mt genomes of *Z. japonica* and *P. iwatensis* and compared them with the genome of other monocotyledons. The results of this study will enhance our understanding of seagrass adaptation to the marine environment and can inform further investigations of the seagrass mt genome.

## Background

Seagrasses are higher marine flowering plants whose ancestors are thought to have been terrestrial monocotyledons that re-adapted to the marine submerged environment through multiple evolutions 70–100 million years ago [1, 2]. Currently, 74 species of seagrasses are known worldwide [3, 4]. Seagrass beds provide habitats for many marine organisms, drive energy flow and material transport of the entire marine ecosystem, and play an important role in regulating global environmental change [1, 5, 6]. Seagrasses have the characteristics and unique structure of both terrestrial and marine plants and can complete their entire life history in the seawater environment, mainly in estuaries, intertidal wetlands of bays, and shallow subtidal waters [7]. Since the environment of seagrasses is highly saline, relatively anoxic, and frequently disturbed by waves and tides, seagrasses have evolved unique morphological structures unknown in terrestrial monocotyledons or freshwater submerged monocotyledons [8, 9]. The emergence of seagrasses represents one of the most striking evolutionary transformations in the evolutionary history of angiosperms. Their unique evolutionary history is one of the most interesting scientific questions in the field of evolutionary biology.

As a member of the “second entry into the sea” club (Like cetaceans in the ocean), seagrass and its evolutionary biology have been a hot topic in recent years. Studies have attempted to reveal the adaptive evolutionary mechanisms of seagrass to the marine environment at the molecular and genomic levels. In 2016, Olsen et al. studied the genome of *Z. marina* for the first time and found that *Z. marina* has lost some genes commonly found in terrestrial angiosperms, such as genes regulating stomatal opening, infrared sensing and ultraviolet protection genes, and terpene and ethylene synthesis genes, and gained some genes regulating ion metabolism, gas exchange, nutrient uptake, and other life activities for adaptation to specific marine environments [10]. In the same year, Lee et al. also found that *Zostera muelleri* had lost or modified multiple genes (such as hormone biosynthesis and signalling genes, and cell wall catabolism genes) compared to terrestrial or floating aquatic plants in order to adapt to the sea [11]. Subsequently, Lee et al. explored the convergent evolutionary features of *Halophila ovalis*, *Z. marina,* and *Z. muelleri* to the marine environment. They found that all three seagrasses lost genes related to ethylene and terpenoid biosynthesis while retaining genes related to salt tolerance [12]. Our previous study showed that the coding sequences of the chloroplast genome of seagrasses have a higher evolutionary rate compared to terrestrial monocotyledons, which may be related to their habitat adaptation [13].

Mitochondria are unique organelles of eukaryotic cells with their own independent genetic system. Because they provide energy for cellular life activities, they are known as the “power house”. The plant mt genome encodes genes related to oxidative phosphorylation and respiratory metabolism, including cytochrome complex I-V subunit genes, cytochrome C synthesis-related genes, ribosomal protein-related genes, tRNAs, rRNAs, and some open reading frames of unknown function [14]. The functional gene sequences of plant mt genomes are very conservative, but the number, location, and order of functional genes vary among species [15]. Adaptive evolution is defined as the improved adaptability of a species to changing environmental conditions during the evolutionary process. Adaptive evolution refers to the ability of a species to adapt well to changing environmental conditions during the evolutionary process, and this process should occur as a distinct change in the sequence of this species. Given the conservation of functional genes in the mt genome, the study of these functional genes will enhance our understanding of the evolution of plant adaptations. To the best of our knowledge, no comparative analysis of the mt genomes of seagrasses adapted to the marine environment has been performed. In this study, we sequenced and annotated the mt genomes of *Z. japonica* and *P. iwatensis* and compared them with the genome of other monocotyledons (which contains all currently published species of Alismatales). Our results will provide additional information for a better understanding of seagrass genetics.

## Results

### Genomic features of Zosteraceae species

**Fig. 1:**
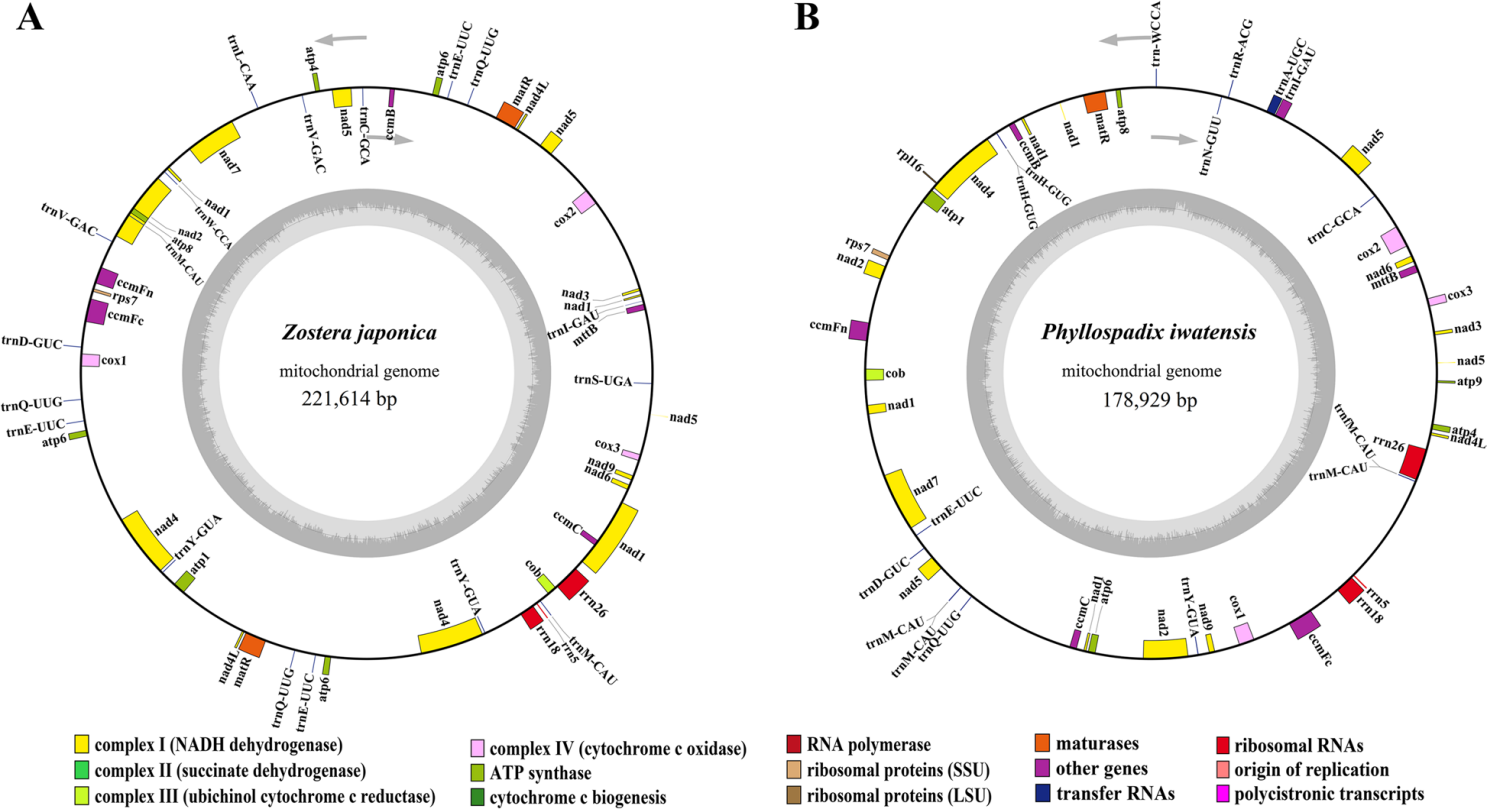
Gene maps of *Z. japonica* and *P. iwatensis* mt genomes

**Fig. 2:**
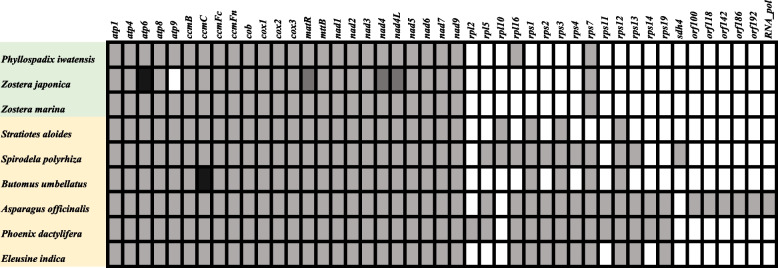
Protein-coding genes present in the mt genomes of the nine species. Dark boxes indicate the presence of three genes, dark grey boxes indicate the presence of two genes, light grey boxes indicate the presence of one gene, and white boxes indicate the absence of the gene. Taxa in green show seagrass, and taxa in yellow show non-marine monocotyledons

**Fig. 3:**
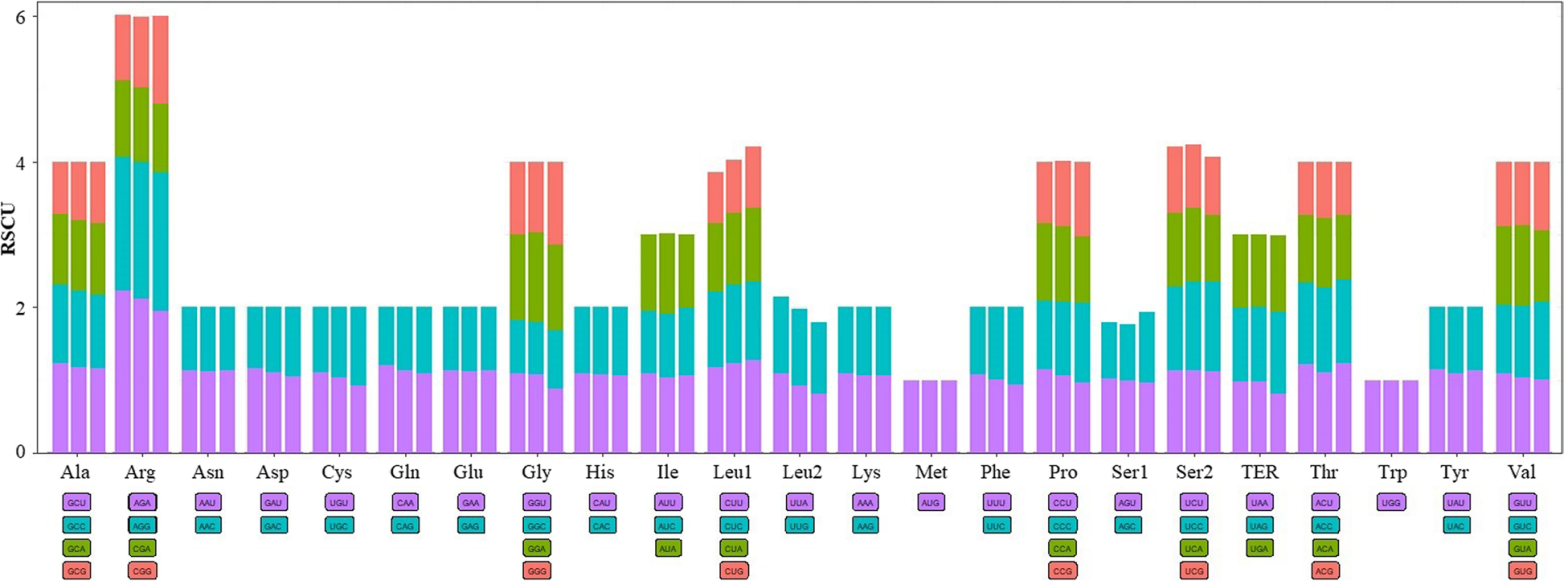
The relative synonymous codon usage (RSCU) of three seagrasses. Each bar chart from left to right is *Z. marina*, *Z. japonica,* and *P. iwatensis*. Codon families are plotted on the X axis

The mt genomes of *Z. japonica* and *P. iwatensis* were circular, while the *Z. marine* was linear. Their lengths were 221,614 (*Z. japonica)*, 178,929 (*P. iwatensis),* and 191,481 (*Z. marine)* (Fig. 1 and Table 1). The highest GC content was in *P. iwatensis* (48.69%), followed by *Z. japonica* (46.26%), and *Z. marina* (45.14%). There are 44, 50, and 38 genes annotated in the *P. iwatensis, Z. japonica,* and *Z. marina* mt genomes, respectively, including 26, 29, and 25 PCGs and 15, 18, and 10 tRNA genes, respectively; and three rRNA genes. The number of PCGs present in the three mt genomes of the *Zosteraceae* species was mostly single-copy PCGs, except for *Z. japonica*, which had three double-copy PCGs (*matR*, *nad4* and *nad4L*) and one triple-copy PCG (*atp6*). Comparing the PCGs of the three seagrasses with three other Alismatales species and three terrestrial monocotyledons, we found that seagrass species have lost many *rpl* genes (except for *rpl16* gene in *P. iwatensis*) and *rps* genes (except for the *rps7* gene) during the evolutionary process (Fig. 2). Analysis of codon usage in the three species showed a strong preference for the ten codon families, Ala, Arg, Gly, Ile, Leu1, Pro, Ser2, Termination codons (TER), Thr, and Val (Fig. 3).

**Table 1:** Basic information on the mt genomes of the nine species in this study

Species	Length (bp)	GC (%)	Total genes	Protein coding genes	tRNA genes	rRNA genes	Accession number
*Phyllospadix iwatensis*	178,929	48.69	44	26	15	3	This study
*Zostera japonica*	221,614	46.26	50	29	18	3	This study
*Zostera marina*	191,481	45.14	38	25	10	3	NC_035345
*Spirodela polyrhiza*	228,493	45.7	59	35	19	3	NC_017840
*Stratiotes aloides*	349,058	45.21	39	28	8	3	NC_035317
*Butomus umbellatus*	450,826	49.1	50	30	16	4	NC_021399
*Asparagus officinalis*	492,062	45.9	59	36	17	6	NC_053642
*Phoenix dactylifera*	715,001	45.14	65	43	18	3	NC_016740
*Eleusine indica*	520,691	43.3	66	34	24	6	NC_040989

### Repeat sequences analysis

**Fig. 4:**
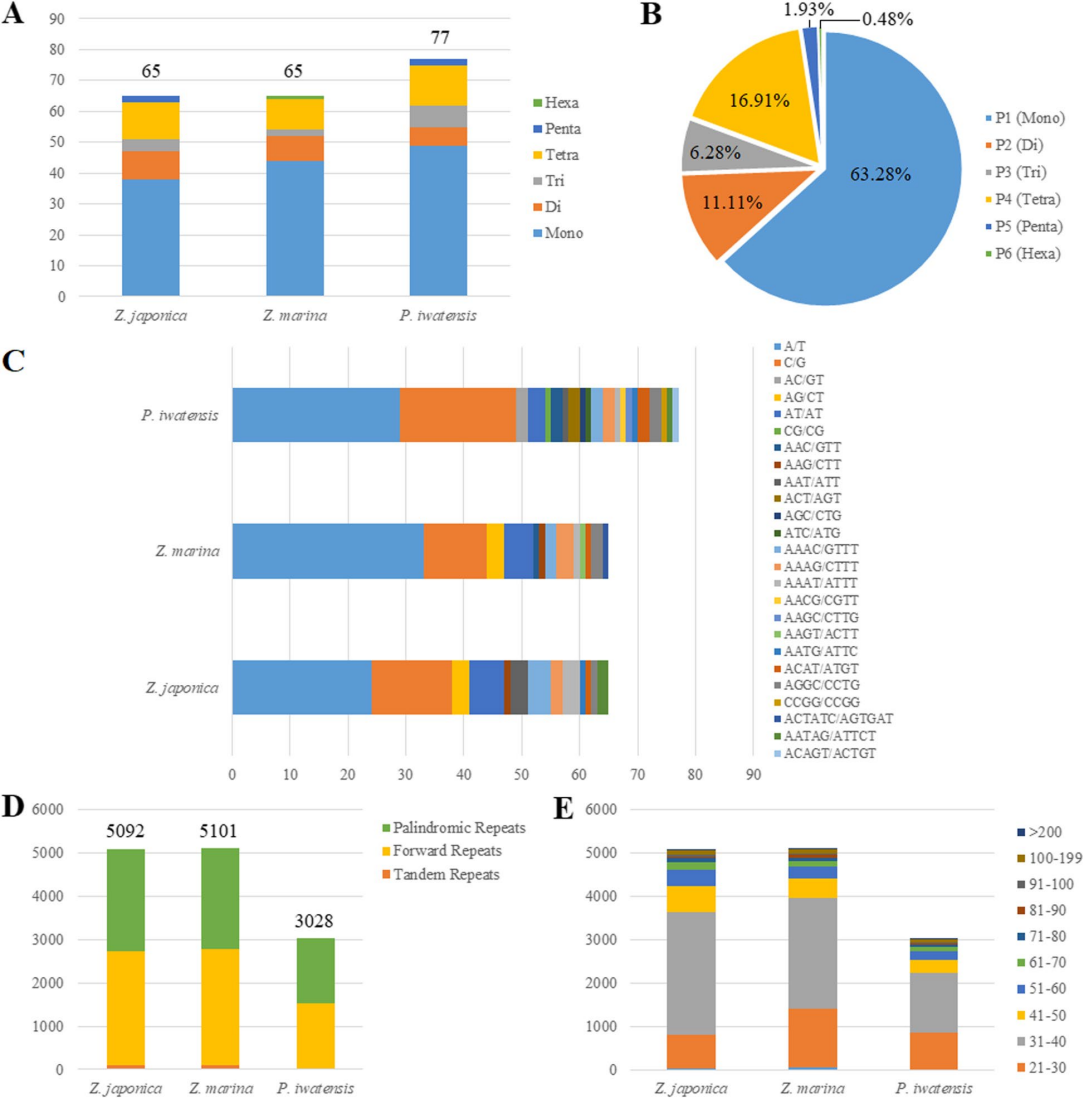
Analysis of repeated sequences of the three seagrass mt genomes compared in this study. **A** The number of SSRs in six SSR types; **B** distribution of SSR types; **C** the number of different SSR repeat unit types; D: the number of repeat elements in three long repeat types; and E: summary of long repeat types by length

A total of 65 (*Z. japonica* and *Z. marina*) to 77 (*P. iwatensis*) SSRs were detected in the three mt genomes, including 38–49 mononucleotides (mono-), 6–9 dinucleotides (di-), 2–7 trinucleotides (tri-), 10–13 tetranucleotides (tetra-), 0–2 pentanucleotide (penta-), and 0–1 hexanucleotide (hexa-) (Fig. 4A). The highest percentage of SSRs was mononucleotides (63.28%), followed by tetranucleotides (16.91%), dinucleotides (11.11%), tetranucleotides (6.28%), pentanucleotides (1.93%), and hexanucleotides (0.48%) (Fig. 4B). Furthermore, we found that the A/T was the most common mononucleotide SSRs type in the three species, and that the repeat units of the other five SSRs were composed mainly of A or T (Fig. 4C). Tandem (T), forward (F), reverse (R), palindromic (P), and complement (C) repeat sequences in the three seagrass mt genomes were identified using a tandem repeat finder and REPuter. We identified three long repeat sequence types in the seagrass mt genome: tandem repeats, forward repeats, and palindromic repeats. We identified 3028 (*P. iwatensis*) to 5101 (*Z. marina*) long repeat elements, of which 19–91 were tandem repeats (Fig. 4D). The lengths of these long repeat sequences were highly variable, with 31–40 bp repeats being the most common among the three repeat types (Fig. 4E).

### Phylogenetic relationships

**Fig. 5:**
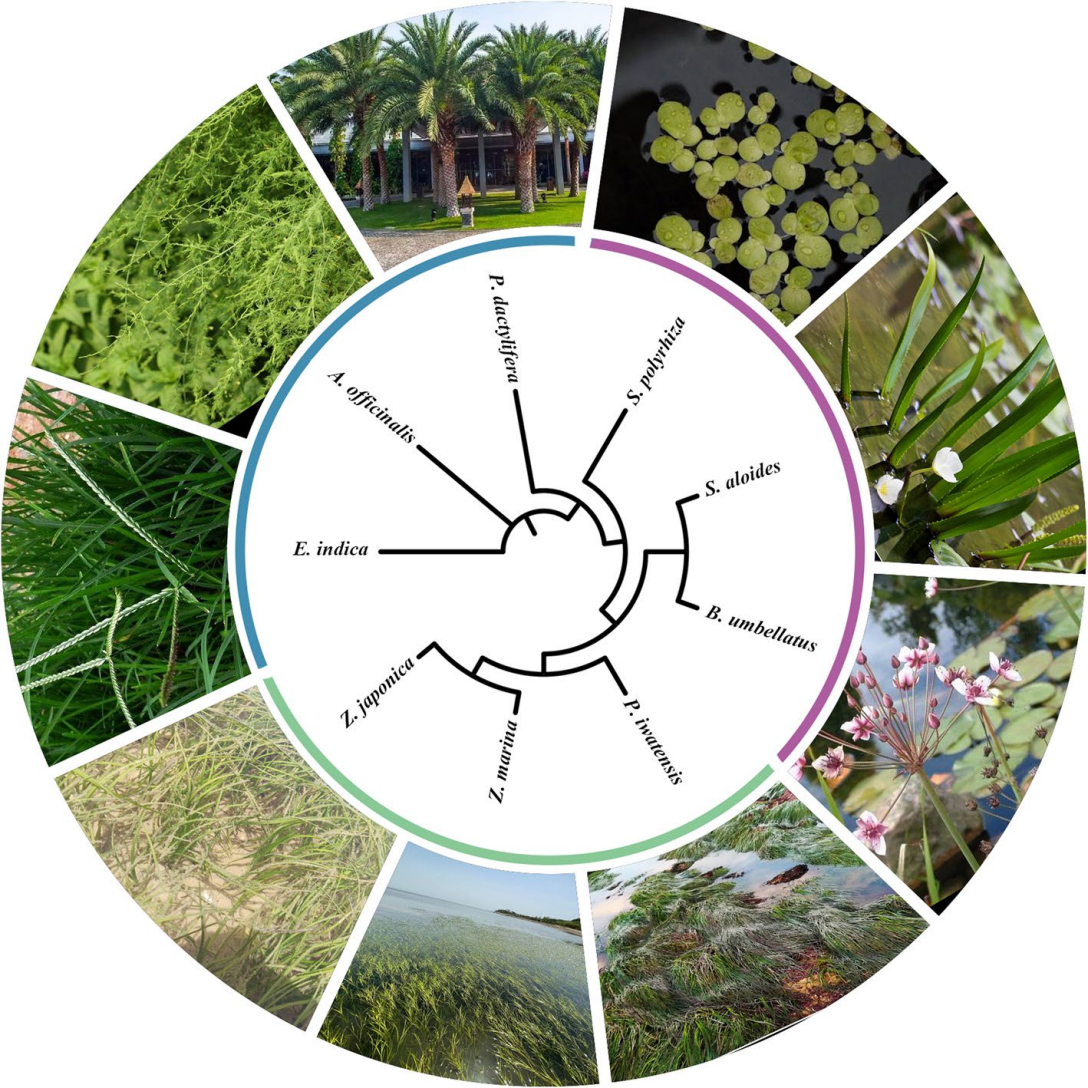
Phylogenetic tree constructed using the maximum likelihood (ML) methods, based on the 23 concatenate protein-coding sequences of whole mt genomes from nine monocotyledons. The green, blue, and purple colors of the arcs between the tree and the picture represent marine, freshwater, and terrestrial species, respectively

To understand the evolutionary status of seagrass mt genomes, a phylogenetic analysis was conducted on *Z. japonica, P. iwatensis,* and seven other monocots species, including four Alismatales (*Z. marina*, *S. polyrhiza*, *S. aloides*, and *B. umbellatus*), one Asparagales (*A. officinalis*), one Arecales (*P. dactylifera*), and one Poales (*E. indica*). A phylogenetic tree was obtained based on the alignment data matrix of 23 shared PCGs (Fig. 5). The phylogenetic tree shows a clear division of the Alismatales and other monocotyledons into two taxa. Regarding Alismatales, the phylogenetic tree shows that *Z. japonica* was a sister species to *Z. marina,* followed by *P. iwatensis*. These results strongly support the theory that marine seagrasses species evolved from the other freshwater species. These data are consistent with the results of a phylogenetic tree based on chloroplast genomes [13].

### Adaptive evolution analysis

**Fig. 6:**
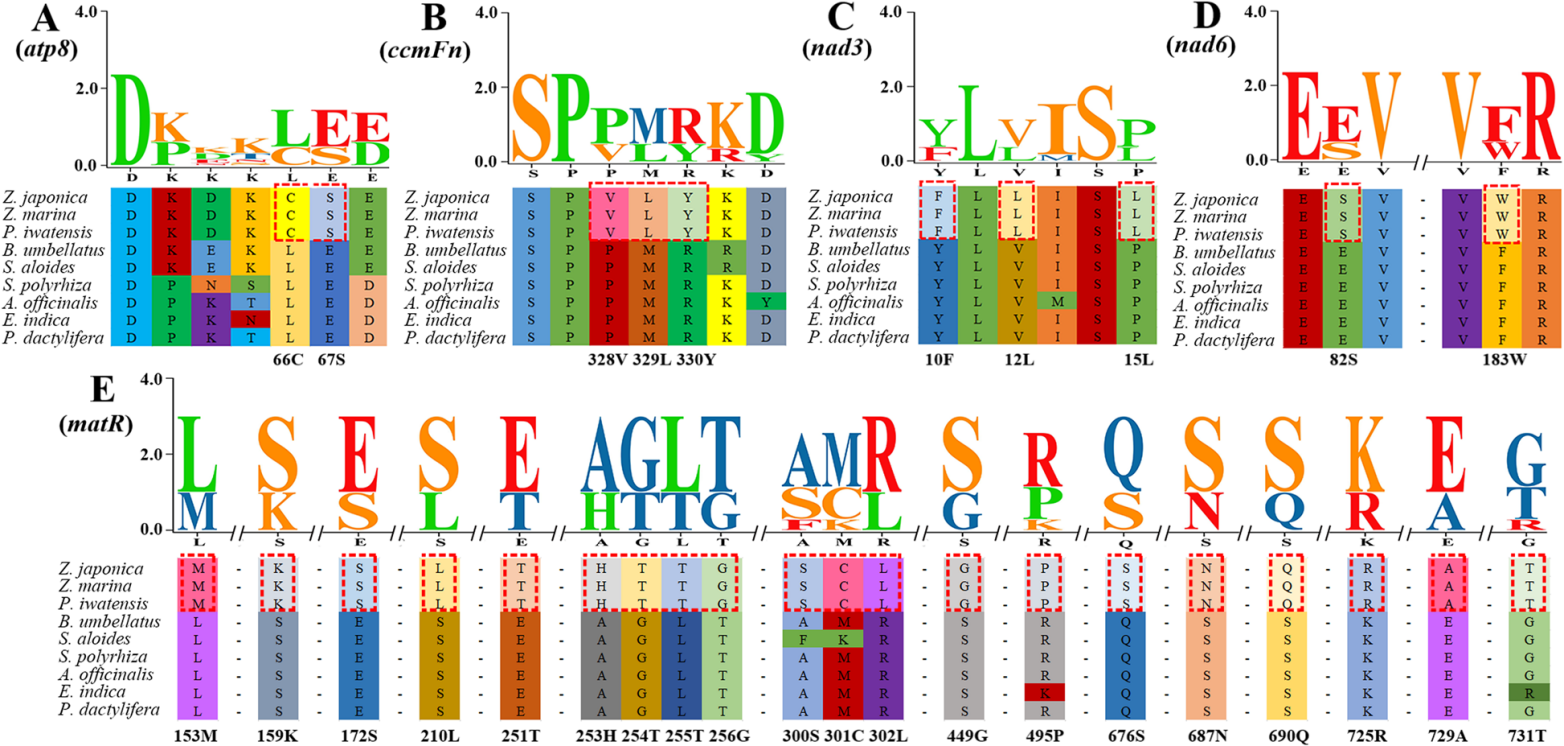
Selection pressure test results of shared protein-coding genes in seagrasses through a branch-site model analysis. The partial alignment of five genes suggesting sites with positive selection in the BEB test. The red blocks stand for the amino acids in seagrass with BEB posterior probability > 0.90. All positively selected sites used the gene location information of *Z. japonica* as the reference sequence

Analysis of the branch model for all shared PCGs showed that the *ω* values (*ω* = 0.41962) was significant (*p* <  0.01), indicating that monocotyledonous plants were strongly purified and selected for during their evolution at the whole-mitochondrion protein level (Table 2). Moreover, although the *ω* values of the seagrass group were lower than those of the terrestrial monocotyledonous group, the evolutionary rate of mt protein-encoding genes in seagrasses did not differ significantly from that of terrestrial monocotyledonous plants (*p* = 0.28). The branch-site model was used to examine the positive selection of individual codons in the seagrass lineage. We found that five genes (*atp8*, *ccmFn*, *nad3*, *nad6*, and *matR*) were positively selected in seagrasses (*p*-value < 0.05) and the BEB posterior probability for 20 amino acid sites larger than 0.90. Among these positively selected genes, the *matR* gene had the highest number of positively selected sites. To better understand the distribution of positive selection in seagrasses, we selected the PCGs of *Z. japonica* as the reference sequence and mapped the above inferred positive selection sites visually to the results of sequence alignment (Fig. 6).

**Table 2:** Selection pressure test results of all shared protein-coding genes in monocotyledons through a Branch model analysis

Model	np	*LnL*	ω for branch	Model compared	*P* − values
A: All branches have same *ω*	18	−52,132.47	ω = 0.41962		
B: All branches have same *ω* = 1	17	−52,426.13	ω = 1	B vs. A	*P* < < 0.01
C: Clade of Seagrass has a *ω*_*1*_; other clade has a *ω*_*2*_	19	−52,131.88	ω_1_ = 0.38859 ω_2_ = 0.42632	C vs. A	*P* = 0.28

## Discussion

### Mitogenome size evolution

**Fig. 7:**
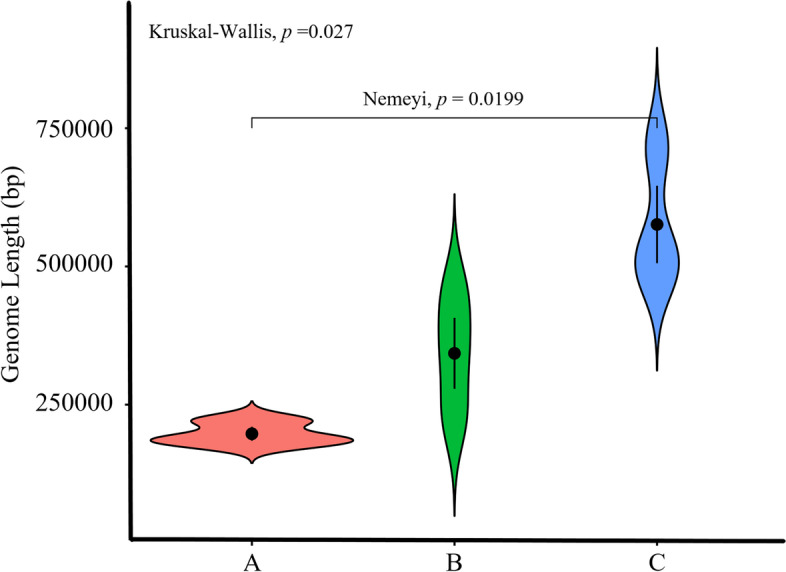
Comparison of mt genome sizes in nine monocotyledonous species. **A** represents marine species including *P. iwatensis*, *Z. japonica,* and *Z. marina*; **B** represents freshwater species including *S. polyrhiza*, *S. aloides,* and *B. umbellatus*; and **C** represents terrestrial species including *A. officinalis*, *P. dactylifera,* and *E. indica*

Mitochondria are organelles with semi-autonomous genetic properties that perform respiratory metabolism to provide energy for life activities. Plant mitochondria possess more complex genomes than animals, with extensive size variations, sequence alignments, repeat content, and highly conserved coding sequences [16]. The circular mt genomes of the two seagrasses (*Z. japonica* and *P. iwatensis*) were sequenced for this study, and the previously published linear mt genome of *Z. marina* was roughly similar in size and gene composition. Their lengths were 221,614 (*Z. japonica)*, 178,929 (*P. iwatensis),* and 191,481 (*Z. marina*). The size of the mt genomes of these three species was smaller than that of other monocotyledons. *P. iwatensis* has the smallest mt genome of all monocotyledons sequenced so far. Previous studies have shown that the relationship between the size of plant mt genomes and their gene numbers exhibits a C-value paradox, i.e., there is no positive correlation between increasing mt genome size and increasing gene numbers [17]. In the present study, we also found gene loss in seagrass species, but the reduced length of the lost genes was not the underlying cause of the reduced mt genome size. It has also been shown that the size of the mt genome is positively influenced by repeat sequences, which could explain why the mt genome of *B. umbellatus,* which had high repeat sequences (approximately 8.3%), was larger than the mt genome of *S. aloides,* which had low repeat sequences (approximately 0.6%) [18, 19]. Surprisingly, the three seagrasses with the smallest mt genomes had the largest number of repeat sequences, so there did not seem to be a clear correlation between the size of the mt genome and the number of repeat sequences. In general, the Alismatales mt genome is relatively small compared with other monocotyledons. Our study determined that the size of mt genomes of monocotyledons in different habitats differed significantly (Kruskal–Wallis, *p* = 0.027), with the mt genomes of marine seagrasses in Alismatales being smaller than those of other freshwater species and significantly smaller than those of other terrestrial monocotyledons (Nemeyi, *p* = 0.0199) (Fig. 7). These results suggest that different habitat types may have an important effect on the mt genome size of monocotyledons. We speculate that during the evolutionary process of “secondary entry into the sea,” seagrasses gradually discarded some “useless sequences” to streamline their mt genomes to better adapt to the marine environment. The GC content of the mt genomes of the three seagrasses ranged from 45.14 to 48.69%, similar to the range of mt genomes in other monocotyledons (Table 1). These results support the conclusion that GC content is conserved in higher plants.

### Mitochondrial protein-coding genes in Seagrasses

Although the functional gene sequences of plant mt genomes are strongly conserved, the number, location, and order of functional genes vary among species [15]. In this study, we found that with the exception of the *rps7* gene and the *rpl16* gene of *P. iwatensis,* seagrass species lost extensive ribosomal protein genes during evolution. A previous survey of mt ribosomal protein genes in 280 angiosperms showed that Alismatales experienced significant gene loss [20]. Our study confirmed this and further found that the loss of ribosomal protein genes was more extensive in seagrasses. The above results seem to imply that ribosomal protein genes in seagrass mt genomes are dispensable. Studies have shown that there is DNA transfer between the organelle genome and the nuclear genome in plants. Where there is DNA sequence transfer between the nuclear genome and the mt genome, chloroplast DNA can be transferred to the mt genome [21, 22]. Whether the ribosomal protein genes missing from the seagrass mt genomes were completely lost or transferred to the nuclear genome was unknown until the complete nuclear genome sequence of *Z. marina* was published, providing evidence that at least four ribosomal protein-coding genes had been transferred to the nucleus [10, 19]. Interestingly, the *rps7* gene is a ribosomal protein gene that is retained by all seagrass species. The reason for this phenomenon may be because the functional transfer to the nucleus is rare [23, 24]. ATP synthase in the plant mt endomembrane, which consists of two parts (F_0_ and F_1_), plays an important role in energy formation [25]. The *atp9* gene codes for a structural protein component of complex V, which is part of the membrane-embedded F_0_ domain. The *atp9* protein, along with *atp8* and *atp6*, contributes to a ring-like structure, forming the channel through which protons diffuse down the mt membrane gradient. This allows ion transport across the membrane against the electrochemical potential gradient, which in turn drives the F1 part of the rotation motor to synthesize the energy molecule ATP [26–28]. Changes in the DNA sequence within the *atp6* and *atp9* genes and their upstream or downstream regions are known to be closely related to male sterility in plants [29, 30]. In our study, we found that the *atp9* gene was lost in *Z. japonica*, whereas three copies of the *atp6* gene existed. We speculate that the *ATP6* gene may have replaced the *ATP9* gene in the mt genome of *Z. japonica* and is performing some of its functions.

### Adaptive evolution

Previous studies have found that the mt genes in seed plants evolved more slowly than their chloroplast genes and nuclear genes [31]. In this study, we found *ω* values less than 1 in all monocotyledons, indicating that the main force affecting the evolution of mt genes was purifying selection. Furthermore, our study found no significant differences in the evolutionary rates of marine seagrasses and other monocotyledons, suggesting that mt PCGs may be conserved among different monocotyledons (*p* > 0.05). Seagrasses grow primarily in the transition zone between marine and terrestrial environments, and as a result, seagrass habitats experience large fluctuations in temperature, salinity, pH, and oxygen concentration. Tidal environments are periodically exposed to the air and susceptible to enhanced UV-B radiation. Previous studies have shown that environmental energy stimulates metabolism at many levels and that energy-rich habitats tend to have higher evolutionary rates [32, 33]. Solar radiation (especially UV radiation) plays a direct role in mutagenesis and may accelerate molecular evolution [34, 35]. Although no significant differences were seen in the evolutionary rates of marine seagrasses and other monocotyledons, the branch-site model analysis showed that 30 sites in the seagrasses lineage on *atp8* (2/30), *ccmFn* (3/30), *nad3* (3/30), *nad6* (2/30), and *matR* (20/30) were under positive selection pressure. This suggested that there were inconsistent selection pressures across sites. Positive selection may provide important functional details related to adaptation to new environments [36]. Previous studies have shown that NADH dehydrogenase is the first as well as the largest complex enzyme in the respiratory chain, while ATP synthase is the last, directly producing ATP [37, 38]. Among them, NADH dehydrogenase acts as a proton pump, so mutations in this complex may affect the efficiency of the proton pump and should affect metabolic efficiency [39, 40]. ATP synthase couples with the electrochemical gradient of the inner mt membrane to synthesize ATP. Variation in these gene sequences may affect the production of ATP [37, 41]. Previous studies have also shown that adaptive evolution of the NADH dehydrogenase complex and ATP synthase play very important roles in the high altitude and intertidal environment adaptation of animals [42–44]. Likewise, seagrasses live in an oxygen-deficient marine environment, which implies an association between gene positive selection and adaptation to energy metabolism in marine environments. In this study, we propose that the positively selected genes *nad3*, *nad6*, and *ATP8* play a role in adaptation to the marine environment in seagrasses. The two remaining positively selected genes were *ccmFn* and *matR*. The *ccmFn* gene primarily synthesizes the N terminus of the cytochrome C maturation protein subunit F and has been reported to increase transcript levels in wheat embryos and seedlings subjected to salt and osmotic stresses [45]. *MatR* is an mt maturase-related gene with the most positive selection sites and has been retained as a conserved ORF in the mtDNAs in nearly all angiosperms [20]. We speculate that these two positively selected genes may also play an important role in enabling seagrasses to adapt to the marine environment.

## Conclusions

The mt genome of two seagrasses, *Z. japonica* and *P. iwatensis*, were assembled and annotated in this study, and extensive analyses were performed based on the DNA sequences and amino acid sequences of the annotated genes. The mt genomes of *Z. japonica* and *P. iwatensis* are circular, while the mt genome of *Z. marine* is linear. The mt genomes of the three seagrasses are smaller than those of other monocotyledons. Repeated sequence analysis showed that the seagrass mt genome contained a large number of repeated sequences. The 31–40 bp repeats were the most abundant long repeat sequences. We also found that seagrass species have lost extensive ribosomal protein genes during evolution, except for the *rps7* gene and the *rpl16* gene of *P. iwatensis*. Phylogenetic results showed that *Z. japonica* was a sister species to *Z. marina,* followed by *P. iwatensis* within Zosteraceae. Finally, we identified five positive selection genes in seagrasses that may be related to their adaptation to the marine environment. These findings will not only help to further investigate the seagrass mt genome, but also be valuable for future studies on the adaptation of seagrasses to the marine environment.

## Materials and methods

### Sample collection and sequencing assembly

The seagrasses *Z. japonica* (120.6801 E, 37.93384 N) and *P. iwatensis* (120.6476 E, 37.97221 N) were collected from collected from Yantai, China, in 2020 and identified by Prof. Xuexi Tang. The *Z. japonica* (voucher number: OUC-S118) and *P. iwatensis* (voucher number: OUC-S119) are currently stored at the Marine Ecology Laboratory of Ocean University of China. The data for *Zostera marina* were downloaded from NCBI (https://www.ncbi.nlm.nih.gov/nuccore/NC_035345.1). Total DNA was extracted from fresh leaves using TRIzol® Reagent (Invitrogen). Total DNA was sequenced using the PacBio Sequel II and an Illumina NovaSeq 6000 platform (150 bp*2) (Shanghai BIOZERON Co., Ltd., Shanghai, China) after library construction. Illumina sequences were assembled using GetOrganelle v1.7.1 [46]. They were compared to PacBio sequences using BWA v0.7.17 [47] in order to extract the trigeneration data of the target samples. The extracted PacBio sequences were mixed with the Illumina sequences to assemble the mt genome using SPAdes v3.14.1 [48]. The assembled mt genomes were polished with clean reads to correct assembled errors using Pilon v1.18 [49]. *Zostera marina* was used as a reference to determine its scaffold start position and orientation to obtain the final mt genome sequences.

### Genome annotation

We compared the protein sequence of *Zostera marina* to the sample genome sequence, to filter out the bad comparison results and remove redundancy. We then ran GeneWise v2.1 [50] for an accurate comparison. Next, we used AUGUSTUS v3.2.2 software [51] to perform De novo gene prediction on the mt genome. Finally, we corrected and integrated the gene set to obtain the sample genome coding genes. The noncoding RNAs (ncRNAs) contained in the genome were predicted using RNAmmer and tRNAscan-SE software [52, 53]. A circular map of the two seagrass species was obtained using Organellar Genome DRAW [54]. The relative synonymous codon usage (RSCU) for all mtDNA of three seagrasses was estimated based on protein-coding genes using CodonW (http://codonw.sourceforge.net/).

### Repeat sequences analysis

Simple sequence repeat (SSR) markers were identified in the three seagrasses sequences using MISA [55] (http://pgrc.ipk-gatersleben.de/misa/misa.html). The repeats of 1, 2, 3, 4, 5, and 6 bases with 8, 5, 4, 3, 3, and 3 repeat numbers, respectively, were identified in this analysis. Forward (F), reverse (R), palindromic (P), and complement (C) repeats were searched using the online program REPuter [56] (https://bibiserv.cebitec.uni-bielefeld.de/reputer) with Hamming distance set to 3 and a minimum repeat size of 30 bp. The tandem repeats with > 6 bp repeat unit were detected using Tandem Repeats Finder v4.09 software [57] (https://tandem.bu.edu/trf/trf.html) with default parameters.

### Phylogenetic construction

In order to determine the phylogenetic position of seagrasses in the Alismatales, we analyzed a total of nine species in this study. Among them, the mt genome sequences of two species (*Z. japonica* and *P. iwatensis*) were newly sequenced, and those of the other Monocotyledons species were downloaded from NCBI database. These sampled Monocotyledons species included all four current species of Alismatales (*Z. marina*, NC_035345; *Spirodela polyrhiza*, NC_017840; *Butomus umbellatus*, NC_021399; and *Stratiotes aloides*, NC_035317), one representative species of Poales (*Eleusine indica*, NC_040989), one representative species of Arecales (*Phoenix dactylifera*, NC_016740), and one representative species of Asparagales (*Asparagus officinalis*, NC_053642). We counted all protein-coding gene sequences (PCGs) of the nine mt genomes. All shared single-copy PCGs were concatenated into a super-matrix and aligned using MUSCLE [58]. The phylogenetic tree was constructed by using the maximum likelihood (ML) method based on the GTRGAMMA model with 1000 bootstrap replicates using RAxML [59]. Finally, iTOL (https://itol.embl.de/login.cgi) was used to visualize and annotate the tree.

### Adaptive evolution analysis

The non-synonymous (*dN*)/synonymous (*dS*) substitution rate (*ω* = *dN/dS*) between single-copy PCGs are usually used to determine the positive Darwinian selection pressure acting on genes. Ratios of *ω* > 1, = 1 and < 1 represent positive selection, neutral selection and negative selection, respectively. The CODEML program implemented in PAML 4.7 package was used to determine positive selection sites or branches [60]. A likelihood ratio test (LRT) with χ2 distribution was performed to determine and compare nested models with a *p* <  0.05 significance threshold. Bayes empirical Bayes (BEB) analysis was used to identify sites under positive selection with Bayesian posterior probability > 0.90 [61].

The branches with seagrasses were used as foreground branches, and the other monocotyledons were the background branches. To determine whether the shared PCGs of seagrasses faced different evolutionary forces in different habitats, we used a null one-ratio model (model = 0, NSsites = 0). This model estimated the same *ω* for all branches against the two-ratio model (model = 2, NSsites = 0), which estimated a variable ω in a specific branch using LRT. A *P*-value < *0.05* was selected to reject the null one-ratio model and evaluate the significance of the alternative hypothesis. We also used a branch-site model in PAML to detect positive selection genes in seagrasses. The LRT was used to contrast a null model Ma0 (model = 2, NSsites = 2, fix-omega = 1, omega = 1) and a model Ma (model = 2, NSsites = 2, fix-omega = 0, omega = 1) of positive selection pressures on the foreground branch. These analyses were based on the ML tree in our study (Fig. 5).

## Data Availability

The datasets generated for this study can be found in National Center for Biotechnology Information (NCBI) under the accession numbers: OP441720 (https://www.ncbi.nlm.nih.gov/nuccore/OP441720) and OP441721 (https://www.ncbi.nlm.nih.gov/nuccore/OP441721).

## Abbreviations

mt: Mitochondrial
RSCU: Relative synonymous codon usage
TER: Termination codons
ncRNA: Noncoding RNAs
SSR: Simple sequence repeat
PCGs: Protein-coding gene sequences
ML: Maximum likelihood
*dN*: Non-synonymous
*dS*: Synonymous
LRT: Likelihood ratio test
BEB: Bayes empirical Bayes
